# Affection of Surgical Decompressive Scale of Optic Canal to Traumatic Optic Neuropathy

**DOI:** 10.3390/brainsci12111442

**Published:** 2022-10-26

**Authors:** Xinyu Li, Zhilin Guo

**Affiliations:** Department of Neurosurgery, Shanghai Ninth People’s Hospital, Shanghai Jiao Tong University School of Medicine, Shanghai 200011, China

**Keywords:** traumatic optic neuropathy, optic canal decompression, efficacy, risk factors

## Abstract

Traumatic optic neuropathy (TON) is damage to the optic nerve that is caused by external violence to the optic nerve during cranial and facial trauma. This kind of injury may result in impaired vision, has a high risk of blindness, and significantly impairs the neurological function of the patient. The treatment of TON is controversial, and many different approaches have been suggested. No one is considered best because the traumatic mechanism is not clear. **Methods:** In this retrospective study, the clinical features of 37 patients diagnosed with TON without light perception who were treated at the Ninth People’s Hospital of Shanghai Jiao Tong University School of Medicine were investigated. A comparison was made between the patient’s visual results before and after therapy. In addition, using a multifactorial linear regression model, the independent risk variables for the degree of improvement in visual acuity (IDVA) following surgery were determined. **Results:** After the operation, 17 people’s visual acuity (VA) was lightless, 20 people’s visual acuity was improved, and 13 people’s visual acuity reached the standard of decerebrate. The efficiency of total optic nerve decompression was 54.1%, and the unblinded rate was 35.1%. Multiple linear regression analysis revealed that fractures of the optic canal and orbit were independent predictors of postoperative VA and IDVA. **Conclusions:** Total optic canal decompression may efficiently and safely enhance the vision of patients who have TON. Patients with TON who do not have fractures of the optic canal and orbit prior to decompression had a more favorable surgical prognosis.

## 1. Introduction

Traumatic optic neuropathy (TON) is acute trauma-induced damage to the optic nerve [1]. Acute or gradual loss of visual acuity (VA) and/or visual field abnormalities of varying degrees following trauma are the primary clinical characteristics associated with TON [2]. The incidence of TON ranges between 0.5% and 5% of all closed head injuries [3,4]. The most common cause of TON are injuries sustained in motor vehicle collisions and falls from great heights. Although there has been improvement in fundamental research, the pathophysiological mechanism is still not well described. The complicated pathophysiology of TON is mostly thought to be caused by injury to the optic nerve caused by a combination of mechanical, chemical, and ischemia factors [5]. The TON injury mechanism may be split into direct and indirect injury [3]. Direct injury can cause severe, irreversible vision loss or even blindness, and its treatment response and prognosis are very poor. However, TON is primarily caused by indirect injury [6]. Indirect injury is caused by open or closed craniomaxillofacial trauma that transfers energy to the optic nerve, which results in optic nerve injury or optic canal fracture.

Observation, high-dose steroid medication, and surgical decompression are the three therapies for TON that have the most acceptance among medical professionals [7]. However, there is a paucity of evidence-based medical evidence that is backed by a significant body of clinical data, choosing the best treatment method is controversy. In recent years, with the continuous advancement of clinical research and microsurgical technique, optic nerve canal decompression surgery has gradually drawn the attention of clinicians [8]. Optic canal decompression surgery alleviates the mechanical compression of the visual pathway by removing the bone around the optic canal, thus providing the enlarged optic nerve with more physical space, enhancing local blood circulation, and preventing nerve injury. The discovery of prognostic indications during the hospital admission of such patients has significant consequences, since these indications may be used to justify medicinal or surgical therapy based on the anticipated result. The management of TON remains contentious. Total optic canal decompression is a new treatment approach that has been proposed at our center in recent years. In this research, we aimed to investigate the safety and efficacy of total optic canal decompression in TON patients with no light perception.

## 2. Materials and Methods

### 2.1. Clinical Data

This study included 37 patients with TON who had undergone total optic canal decompression (TOCD) in the Department of Neurosurgery of the Ninth People’s Hospital of Shanghai Jiao Tong University School of Medicine from January 2018 to December 2021. All patients included in the study were treated by the same neurosurgeon for TOCD. There were 34 males and 3 females. The age range was 19–67 years, with an average age of 37.8 ± 13.6 years. The informed consent form and surgical consent form were signed by all patients and their families before surgery. This study is in accordance with the Declaration of Helsinki.

### 2.2. Inclusion and Exclusion Criteria

Inclusion criteria: (1) TOCD was performed; (2) presenting with a definite history of craniocerebral trauma; (3) clear vision loss or visual impairment by specialist examination; (4) exclusion of visual impairment due to previous disease disorders; (5) the relative afferent pupillary defect (RAPD) was positive.

Exclusion criteria: (1) total loss of consciousness; (2) examination suggests significant optic nerve dissection; (3) possibility of internal carotid artery dissection or internal carotid artery pseudoaneurysm or internal carotid cavernous sinus fistula.

### 2.3. Study Methods

Age, gender, VA before treatment, VA at 7 days after surgery, time to medical treatment, and additional problems, such as ethmoid or sphenoid sinus hematoma, orbital fracture (OF), and optic canal fracture (OCF), were documented for each of the 37 patients. The efficacy of TOCD for TON patients in our center was calculated. A multi-factor linear regression analysis of the above clinical indicators was also performed to screen the influencing factors affecting the recovery of VA and IDVA in TON patients. VA was measured with the Snellen Chart. For the purpose of conducting a study based on an extended scale to determine the impact of TOCD, VA were transformed into the logarithm of the minimum angle of resolution (logMAR) unit (Table 1).

### 2.4. Efficacy Assessment

The prognosis of clinical efficacy was based on the final VA of patients at follow-up, and VA was divided into different grades in this study: 0: No light perception (NLP), 1: light perception (LP), 2: hand motion (HM), 3: finger counting (FC), and 4: above FC. An improvement of one level of postoperative VA compared to preoperative VA is defined as improvement in VA, and an improvement of two levels is defined as unblinded. VA improvement rate defined as: number of people with improved vision after surgery/total number of patients. Improvement degree of VA IDVA = (Final LogMAR VA − preoperative LogMAR VA)/(0.12 × −preoperative LogMAR VA) [9].

### 2.5. Surgical Procedure

All surgery was performed by a single experienced surgeon (Dr. Zhilin Guo), under general anesthesia. Upon admission, all patients received intravenous methylprednisolone 500 mg/day and oral metocobalamin. Due to the prevalence of traumatic brain injury, decompression of the optic canal in TON patients is performed with the microneurosurgical technique. The lateral sphenoid ridge was then resected to the level of the superior orbital fissure with the help of Stryker high speed drill. The anterior clinoid process, including optic canal roof and optic strut, was removed with a diamond drill, and the falciform ligament and optic nerve sheath were then divided from the optic foramen to the annulus of Zinn. Hemostasis was completed with a gelatin sponge, with no or less use of electrocautery as possible as gentamicin-saline was used to flush the wound. After the operation, high-dose intravenous methylprednisolone was given for three days, and intravenous ceftriaxone was given to prevent infection.

### 2.6. Statistical Analysis

Statistics was evaluated using SPSS version 24 (SPSS Inc., Chicago, IL, USA). The counting data were expressed in terms of individuals and percentages (%) and measurement data were expressed as mean ± standard deviation (SD). The improvement rate of VA was compared by χ^2^ test and Fisher’s exact test [10]. Differences less than 0.05 (*p* < 0.05) were considered statistically significant.

## 3. Results

### 3.1. The Baseline Data of the Patients

Of the 37 TON patients, 21 cases affected the right eye while 16 cases affected the left eye and 24 were injured by car accidents, 10 by falls, two by heavy objects, and one by explosions (Table 2). Preoperative CT examination revealed OF in 31 cases, OCF in eight cases, and ethmoid or sphenoid sinus hematoma in six cases. The entrance Glasgow Coma Scale (GCS) score was 9–12. The period between injury and operation was 1–30 days, with an average of 3.12 days, including 30 patients with operations performed within three days following injury. The average intraoperative bleeding volume was 526 mL.

### 3.2. Overall VA Improvement and IDVA

**Figure 1:**
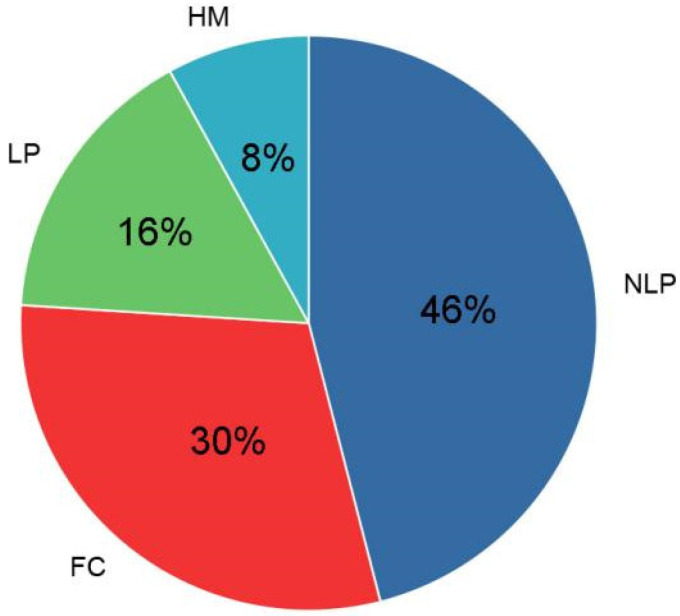
Recovery of vision in TON patients after surgery. NLP: No light perception; LP: Light perception; HM: Hand movement; FC: Finger counting.

VA after surgery ranged from −5.0 to −2, with a mean value of −3.78 ± 1.30. IDVA ranged from 0 to 0.58. Postoperative VA was NLP in 17 people, and six patients had their VA restored to LP. Thirteen people reached the standard of unblinded (Figure 1). The efficiency of TOCD was 54.1%, and the unblinded rate was 35.1%. 

### 3.3. Multiple Linear Regression Analysis of Postoperative VA and the IDVA

In the multiple linear regression analysis model, OCF and OF were significantly associated with postoperative VA (*p* < 0.05), and both were independent risk factors for postoperative visual prognosis (Table 3). Moreover, OCF and OF showed a significant correlation with IDVA (Table 4). In TON patients with OCF, the effective rate was 50% compared to 55.2% in TON patients without OCF, with a statistical difference between the two groups (*p* < 0.05).

### 3.4. Surgical Complications

All patients had no postoperative complications, such as cerebrospinal fluid nasal leakage, intraorbital infection, or intracranial infection. There was no postoperative worsening of VA in any of the TON patients.

## 4. Discussion

TON is a serious complication after craniocerebral injury [9]. Although TON has been extensively researched, the exact pathological mechanism of this disease is still unclear. Increased pressure in the optic canal after the injury is considered to be the main pathophysiological manifestation of optic nerve injury [11]. Other studies suggest that axonal damage occurs after optic nerve injury and the number decreases dramatically [12]. The increased intracanalicular pressure after optic nerve injury may trigger a series of molecular and chemical changes, causing a cascade of molecular and chemical mediators that cause optic nerve cells to initiate the apoptotic process [13,14]. Moreover, the occurrence of TON is often associated with OCF, where fracture fragments can directly cause damage to the optic nerve as well as compression and rupture of the vessels in the canal, which can also affect the vascular supply to the optic nerve and affect the prognosis [14]. There is a lack of valid evidence-based medical evidence regarding treatment options for patients with TON. According to findings from earlier research, the efficiency of steroid treatment was 4.3–44%, while the efficiency of surgery combined with steroid was 60.9–71.1% [2,15,16]. A growing number of experts believe that TON treated with aggressive surgical treatment may provide a good long-term prognosis compared to traditional conservative treatment [17,18,19]. Therefore, adequate and complete decompression of the optic nerve is key to the patient’s prognosis.

Most current studies generally use a surgical approach with a decompression range of half the circumference of the optic canal wall, while fewer studies have been performed on TOCD. The aim of this study was to investigate the effectiveness of TOCD in the treatment of TON and to analyze the relationship between different clinical variables and the improvement of postoperative VA. Our study included a total of 37 participants, ranging in age from 19 to 67 years old. According to our research, being in a car accident was the leading cause of ITON, followed by experiencing a fall. In the present study, the efficiency of TOCD was 54.1%, and the unblinded rate was 35.1%. In a prior retrospective study evaluating the safety and efficacy of endoscopic optic nerve decompression for TON patients with NLP, the VA improvement rate was 46.9% [13]. This indicates that the efficiency of TOCD is higher than that of endoscopic optic nerve decompression. 

Patients who have TON can benefit from the endoscopic endonasal optic nerve decompression technique, an effective, and minimally invasive technique [20,21]. However, the disadvantages of endoscopic optic nerve decompression are the small decompression range (range about 168°) and the risk of damage to the internal carotid artery [22]. We hypothesize that the explanation for this difference is that TOCD is better able to make space for the nerve to swell while simultaneously reducing the harmful effect of compression and reestablishing nerve function. Compared to endoscopic optic nerve decompression, TOCD provides an adequate operating area and a familiar perspective. Further prospectively designed and large cohort studies are needed to compare the clinical outcomes of TOCF or endonasal optic nerve decompression.

According to the results of our research, both OCF and CF can be considered independent prognostic factors for VA. Those with OCF showed a decreased IDVA compared to patients without OCF. We think that this is mostly due to the irreparable damage to the optic nerve caused by OCF, as well as the production of a hematoma, which increases pressure in the optic canal. Therefore, we recommend prompt surgical treatment once OCF is detected. This result is consistent with prior research [23,24]. Some studies have found the opposite, with the presence or absence of OCF not making a significant difference in the outcome of patients with TON [25,26]. The existence of OF is most typically induced by large external violent impacts and the trauma that generates OF may also be passed to the optic nerve, producing equivalent damage and compromising the prognosis of vision in TON patients. On the other hand, some studies have concluded that OF is not significantly related to the visual prognosis of TON [24]. More studies are required to conclusively demonstrate the connection between OF, OCF, and optic nerve injury.

In our research, we could not find any evidence of a correlation between time to surgical treatment and prognosis (VA prognosis and IDVA). This conclusion could be due to the small sample of subjects. There was still a lot of debate over when surgical intervention should take place [27]. Some studies advocate early intervention. Others have noticed that even with delayed surgery, vision will also improve [28]. Many current studies have shown that patients who undergo surgery within seven days have a better prognosis [29]. It is possible that timing is not an independent factor in determining the prognosis of eyesight in patients with TON. There is a continuing need for large prospective studies to obtain further data regarding the ideal date. In addition, vision after surgery is dependent not only on the time of the operation, but also on the degree and kind of nerve injury that was caused. Even if TON patients with NLP are delayed in treatment, we recommended doctors to never give up on them.

Ethmoid or sphenoid sinus hematoma has been reported as a risk factor for postoperative visual prognosis after TON and is recommended to be removed during surgery, but the exact mechanism by which it affects final visual acuity is not known [26]. No relationship between ethmoid or sphenoid sinus hematoma and prognosis was found in the current study. We suspect that this may be due to the fact that ethmoid or sphenoid sinus hematoma are not severe enough to damage the optic nerve. 

## 5. Conclusions

For patients suffering from TON with NLP, TOCD is a safe and effective treatment modality. Patients with TON who delay treatment still have the possibility of a better prognosis, so TOCD should be performed as soon as possible after a thorough examination of the patient. To further demonstrate the efficacy of TOCD for patients with ITON and to determine the precise surgical indication, large-sample, controlled, prospective studies are required.

## Figures and Tables

**Table 1:** Relationship between visual acuity and logarithm of the minimum angle of resolution (logMAR) visual acuity chart.

Visual Acuity	logMAR Units
NLP	−5.0
LP	−4.0
HM	−3.0
FC	−2.0
0.01	−2.0
0.03	−1.6
0.05	−1.3
0.06	−1.2
0.07	−1.18
0.08	−1.10
0.1	−1.0
0.16	−0.8
0.2	−0.7
0.25	−0.6
0.32	−0.5
0.4	−0.4
0.5	−0.3
0.67	−0.18
0.8	−0.1
1.0	0

**Table 2:** Clinical demographic data of the patients.

**Characteristic**	**Case** **(%)**
**Gender**	
male	34 (91.9%)
Female	3 (8.1 %)
**Injury type**	
car accidents	24 (64.8%)
falls	10 (27.1%)
heavy objects	2 (5.4%)
explosions	1 (2.7 %)
**Timing of surgery**	
≤1days	21(56.8%)
1-3 days	9 (24.3%)
3-7 days	3 (8.1%)
>7 days	4 (10.8%)
**Blood accumulation in the septal sinus/pars sinus**	
Yes	6 (16.2%)
NO	31(83.8%)
OCF	
Yes	8 (21.6%)
No	29(78.4%)
**Orbital fractures**	
Yes	31 (83.8%)
No	6 (16.2%)

**Table 3:** Multiple linear regression analysis of postoperative visual acuity in patients with TON.

Variables	Coefficient	SE	t	*P*
Intercept	3.88	0.77	5.03	2.15 × 10^−5^
OCF	−1.40	0.45	−3.13	0.004
CF	−1.15	0.45	−2.55	0.02
Blood accumulation in the septal sinus/pars sinus	0.56	0.44	1.28	0.21
Age	−0.004	0.02	−0.30	0.77
Timing of surgery	0.002	0.03	0.08	0.94
Gender	−1.24	0.66	1.88	0.07

**Table 4:** Multiple linear regression analysis of IDVA in patients with TON.

Variables	Coefficient	SE	t	*P*
Intercept	0.76	0.15	4.93	2.83 × 10^−5^
OCF	−0.27	0.08	−3.14	0.004
CF	−0.23	0.09	−2.57	0.02
Blood accumulation in the septal sinus/pars sinus	0.11	0.08	1.37	0.21
Age	−0.009	0.003	−0.26	0.77
Timing of surgery	0.004	0.005	0.07	0.94
Gender	−0.24	0.13	1.84	0.07

## Data Availability

Data and materials will be available upon request.
